# Interventions Based on Social Norms Could Benefit From Considering Adversarial Information Environments: Comment on Constantino et al. (2022)

**DOI:** 10.1177/15291006221114132

**Published:** 2022-10-13

**Authors:** Stephan Lewandowsky, Sander van der Linden

**Affiliations:** 1 School of Psychological Science, University of Bristol; 2 School of Psychological Science, University of Western Australia; 3 Department of Psychology, University of Cambridge

In 1859, John Tyndall presented his findings to the Royal Society about a perfectly colorless and odorless gas, known as “carbonic acid,” that he had discovered to be nearly opaque to radiant heat despite being transparent to visible light. We now refer to carbonic acid as carbon dioxide (CO_2_), and before the end of the 19th century, a Swedish physicist had already identified its potential to alter the Earth’s climate as a result of the combustion of fossil fuels (Arrhenius, 1896). More than 120 years later, scientists continue to warn the world about the adverse effects of CO_2_ emissions (Intergovernmental Panel on Climate Change [IPCC], 2022), but to date the policy response has been inadequate, and we are on track to exceed purportedly “safe” global temperature increases (IPCC, 2022). There are many reasons for our collective failure to respond adequately to climate change, ranging from well-organized political opposition (Lewandowsky, 2021) to the inherent sociopsychological challenges posed by a problem that requires global collective action and large-scale behavior change by millions of people around the world (e.g., Smith & Mayer, 2018).

The article by Constantino and colleagues focuses on the role of social norms in facilitating the widespread shift in behavioral practices that is required to deal with climate change. Humans are social animals and hence sensitive to perceived social norms: We tend to engage in behaviors on the basis of expectations of what others around us do or think what should be done. When those norms change, people’s behaviors also change. The key point made by Constantino and colleagues is that localized interventions can incentivize change in a subset of a population, creating minorities committed to a prosocial or proenvironmental nonnormative belief or behavior. The tendency to conform, in turn, leads others to adopt this nonnormative behavior, which begins to spread through social networks. Once a critical mass has adopted the nonnormative behavior, these social dynamics trigger abrupt, widespread, and nonlinear change, eventually tipping societies toward more sustainable equilibria (p. 51).

Framed within this overarching approach, Constantino and colleagues provide admirably detailed insights into how those large-scale changes can be triggered through local interventions. An illustrative case involves the spread of solar panels across Germany during the early 2000s: It was initially observed that in communities in which a small group of early adaptors were in close proximity (e.g., in the same street), local cascades were triggered that relatively quickly created communities in which people *without* solar panels were in the minority. These local clusters, however, failed to spread into neighboring communities until policy makers launched a “100,000 Roofs” program that provided reduced-interest loans and other incentives to create bridges into neighboring communities to trigger further local cascades. By 2016, German citizens were generating more solar electricity per capita than any other country in the world.

Constantino and colleagues argue that social norms can assist with several different aspects of the climate-change problem, ranging from social equity and power asymmetries (i.e., through norms around inclusivity, fairness, distributive justice) to culture and identity (e.g., norms relating to meat consumption may facilitate lifestyle changes) and unduly steep future discounting (i.e., through injunctive norms specifying what “ought” to be done). Constantino and colleagues also recognize the importance of abstract “meta” norms, such as the current focus on individualism at the expense of the common good in many Western democracies. Overall, Constantino and colleagues provide a highly detailed and nuanced overview of the role of social norms and their limitations. Here, we expand and shift focus on some of their discussion by viewing norms through the lens of the adversarial environment that surrounds climate change. We also draw connections to a closely related issue—namely, communication of the scientific consensus on climate change—that has been shown to be useful in adversarial environments.

## Social Norms in an Adversarial Environment

The complexity of climate change and the difficulties associated with global coordination of a response are only partly responsible for our collective inability to forcefully address the problem. A large share of the blame rests with the organized opposition to climate mitigation. There is no doubt that disinformation about climate change is disseminated in an organized and well-funded manner. The annual budget (2003–2010) of think tanks that are known to be involved in creating and disseminating climate disinformation was around $900 million (Brulle, 2013). Moreover, between 2000 and 2016, more than $2 billion was spent by Congressional lobbyists to oppose climate legislation (Brulle, 2018); most of that money was expended on legislators with an antienvironmental track record (Goldberg, Marlon, et al., 2020). Two major legislations aimed at mitigating climate change, the Lieberman-McCain Climate Stewardship Act (2007) and the Waxman-Markey American Clean Energy and Security Act (2009), failed during this period. Any efforts to address climate change thus take place in an adversarial environment in which contrarian actors will seek to counteract and undermine any initiatives aimed at climate mitigation.

There are several ways in which interventions based on social norms may be jeopardized in an adversarial environment. It is important to highlight those risks to provide additional context for the approach explored by Constantino and colleagues.

First, as Constantino and colleagues already note, people’s reliance on norms and their tendency for conformity is a double-edged sword: Although conformity may assist with behavior change if norms are changing, it can also lead as entrenchment of the status quo if people perceive norms to be unchanging. Indeed, appealing to the prevailing majority behavior is counterproductive if that behavior is damaging to the climate. Under those circumstances, communicators can appeal to a dynamic shift in a norm (“people’s food preferences are becoming more sustainable”), but such appeals are more difficult to sustain in the face of countermessages that endorse the status quo (“nine out of 10 Americans prefer meat in their diet”). Given that people demonstrate a persistent bias for the status quo (Samuelson & Zeckhauser, 1988), political campaigns can readily facilitate a preference for the status quo and thereby detract from generating support for new proenvironmental policies (Bolsen et al., 2014).

Second, the effectiveness of social-norm interventions rests on the *perceived* prevalence of others’ actions. If people’s perceptions of prevalence deviate from the actual prevalence, norm-based interventions are difficult and may even backfire. In the context of climate change, there are several reports of a major divergence between the perceived prevalence and the actual prevalence of behaviors. For example, in an Australian study, the small share of people (barely over 5%) who denied that climate change was occurring thought that their minority views were shared by more than 40% of the population, an overestimate known as a *false-consensus effect*. Conversely, the majority of respondents (> 50%) who correctly identified human causes for climate change thought that their opinion was shared by only 40% of others, an underestimate known as *pluralistic ignorance* (Leviston et al., 2013). Similar effects have been reported by Lewandowsky et al. (2021), Mildenberger and Tingley (2019), and Pearson et al. (2018). The divergence between perceived and actual opinions can have several profound implications, ranging from people’s reluctance to express their concern if they (falsely) believe that others do not share it (Geiger & Swim, 2016) to resistance to persuasion if people mistakenly think that their minority views are widely shared (e.g., Suls et al., 1988). Moreover, in the long run, people tend to shift their attitudes or behaviors in the direction of what they perceive to be the prevailing majority opinion (even if it is not; Botvin et al., 1992; Eisner et al., 2020; Prentice & Miller, 1993), which may translate into an erosion of support for climate mitigation by the majority who accept the science if they believe to be in the minority. Pluralistic ignorance thus creates an ironic bifurcation for social-norm approaches: On the one hand, a naive appeal to social norms risks backfiring if people misperceive the norm and are therefore distrustful of the information. On the other hand, as noted by Constantino and colleagues, the very existence of pluralistic ignorance calls for interventions that inform people of the actual opinion landscape to eliminate the adverse fallouts of pluralistic ignorance (see Lewandowsky et al., 2021).

Third, norms can unravel very quickly when key events signal a realignment in public attitudes. This can have socially advantageous consequences, as illustrated by the rapid increase in the public’s perception of a social norm in support of gay marriage after the Supreme Court ruled in favor of same-sex marriage (Tankard & Paluck, 2017). Unfortunately, rapid shifts in norms can also have deleterious consequences, as in the case of the election of Donald Trump in 2016, which was widely taken to legitimize the expression of xenophobic attitudes that previously had been deemed unacceptable (Bursztyn et al., 2017). The “racist contagion” that followed Trump’s election was detectable on the other side of the Atlantic, in the 13 countries that are sampled for the European Social Survey (Giani & Méon, 2021). Moreover, Donald Trump’s refusal to concede his election loss in 2020 sparked false-consensus effects around a nonpeaceful transfer of power (Weinschenk et al., 2021). Another example comes from the COVID-19 pandemic, when local norms around mask wearing differed widely such that mask wearing declined in neighborhoods with a larger relative share of Republicans (Baxter-King et al., 2022). In the context of climate change, any social-norm intervention may therefore also be upended by unexpected key events or local disturbances, suggesting that alternative strategies to cope with such events should be put in place. We present one such alternative strategy below.

Finally, social-norm interventions must also consider research showing that not everyone is equally “nudgeable.” Constantino and colleagues touch on several variables that determine nudgeability. Here, we explore the role of political ideology further. In one field study, providing feedback to households on their energy consumption relative to their neighbors was up to 4 times more effective with political liberals than with conservatives (Costa & Kahn, 2013). Conservatives were more likely to opt out of receiving reports on their energy consumptions and were considerably more likely (compared with liberals) to indicate that they disliked the “nudging” information. However, it does not necessarily follow that conservatives are always impervious to “nudges” or norm-based interventions. Quite to the contrary, conservatives have been shown to have a greater desire for a shared reality and conformity with their in-group than liberals (Jost et al., 2018). In the climate-change context, Goldberg, van der Linden, et al. (2020) showed that conservatives’ attitudes toward climate change were strongly associated with their perceived social consensus among friends and family. The more that respondents thought that their friends and family accepted climate change and were making efforts to mitigate climate change, the more they themselves acknowledged the existence and causes of climate change. It turns out that the perception of a consensus in a relevant reference group is a particularly powerful norm that has often been shown to be effective across partisan lines.

## Building on Norms: Social and Scientific Consensus

A body of recent research in the cognition of climate change has examined the role of the perceived consensus among a specified reference group—in particular, among scientists—in shaping people’s attitudes toward climate change. This research is characterized by specifying a specific reference group and, frequently, providing exact information about the level of consensus within that reference group.

The reference group can take a number of forms: Whereas Goldberg, van der Linden, et al. (2020) probed people’s perceptions of attitudes among friends and family, other researchers have (a) queried the perceived consensus among readers of a blog on the basis of reader comments (Lewandowsky et al., 2019) or (b) provided information about the actual scientific consensus on climate change (e.g., Lewandowsky et al., 2013). The latter intervention seems to be particularly promising because it has been shown repeatedly that providing information about the scientific consensus can shift people’s attitudes about climate change and, in several cases, has reduced the polarization along partisan lines (see Cook et al., 2017; Goldberg, van der Linden, Ballew, et al., 2019; Imundo & Rapp, 2022; Lewandowsky et al., 2013; van der Linden et al., 2018). The broad applicability of consensus messaging was recently underscored by a large field study in the Czech Republic that showed that COVID-19 vaccine uptake significantly increased in a group that was provided information about the consensual trust in the vaccine by Czech doctors (90% of nearly 10,000 doctors sampled trusted the vaccines) compared with a control group (Bartoš et al., 2022).

In climate change, people’s perceptions of what scientists believe has been identified as a key “gateway” cognition to attitude change about climate change (for a review, see van der Linden, 2021). In consensus experiments, the treatment group is typically provided with a descriptive norm about the scientific consensus; for example, it has been estimated that 97% of climate scientists agree on the fundamental causes of climate change (Cook et al., 2016). In comparison to a no-intervention control group, people who received the consensus information are typically more likely to accept the existence of climate change and its human causes and, in turn, to support policy interventions (Brewer & McKnight, 2017; Goldberg, van der Linden, Ballew, et al., 2019; Harris et al., 2018; Imundo & Rapp, 2022; Kerr & Wilson, 2018; Lewandowsky et al., 2013; Myers et al., 2015; van der Linden et al., 2015, 2019). Two recent meta-analyses have further bolstered the power of highlighting consensus among climate experts (see Rode et al., 2021; van Stekelenburg et al., 2022), finding significant average effect sizes on perceived consensus (Hedges’s *g* = 0.56; van Stekelenburg et al., 2022) and on private attitudes (Hedges’s *g* = 0.09; Rode et al., 2021; Hedges’s *g* = 0.12; van Stekelenburg et al., 2022).

Consensus information has been shown to be effective among people who are skeptical of climate change (Bolsen et al., 2021; Lewandowsky et al., 2013; van der Linden et al., 2018), and it has been shown to at least partially neutralize misinformation and conspiracy theories about climate change being a hoax (Bolsen et al., 2021; Cook et al., 2017; van der Linden et al., 2017). Although they are an out-group, nonpartisan experts can be effective communicators in politicized debates (Flores et al., 2022). Moreover, the effect of communicating the scientific consensus on climate change can further be enhanced by having the consensus presented by prototypical in-group members such as Republicans speaking out against their partisan interests (Benegal & Scruggs, 2018). Learning about the scientific consensus can also help reduce the spiral of silence by encouraging conversations about climate change with other people in one’s social network (Goldberg, van der Linden, Maibach & Leiserowitz, 2019).

The importance of consensus messaging is also, somewhat ironically, underscored by contrarian attempts to undermine the scientific consensus. For example, one analysis of conservative op-eds by syndicated columnists found that the most common argument was the erroneous claim that there was no scientific consensus (Elsasser & Dunlap, 2013). Likewise, the most shared climate article on social media in 2016 claimed that there was no scientific consensus (Readfearn, 2016); the claim was based on a meaningless Internet petition that collected signatures of “scientists” against the consensus, but few signatories had any relevant scientific credentials. This petition was found to be the most damaging in reducing acceptance of climate change in an experiment comparing six common myths (van der Linden et al., 2017). Fortunately, however, several studies have shown that attempts to undermine the consensus can be effectively neutralized by “inoculation”; that is, by forewarning participants about similar efforts by the tobacco industry to undermine medical science by appealing to “fake experts” to create the chimerical appearance of a debate (Cook et al., 2017; see also van der Linden et al., 2017). Thus, although consensus messaging is subject to disruption by adversaries, it has also been shown to be resilient to such attempts when it is additionally accompanied by anticipatory countermeasures (Lewandowsky & Van Der Linden, 2021).

As mentioned earlier, the challenge with static descriptive norms is that communicating them works only if there is a consensus to leverage. Sometimes, for example, there is no group consensus, or the prevailing consensus (e.g., meat eating) might even run counter to efforts to change population behavior. In contrast, highlighting scientific consensus benefits from several unique features: (a) It leverages the wisdom of crowds by aggregating the opinions of thousands of independent experts (Budescu & Chen, 2015), which—as a decision heuristic—people prefer to use over random crowds (Mannes et al., 2014); (b) it includes an appeal to expert authority which can enhance its persuasiveness (Cialdini et al., 2015; Flores et al., 2022); (c) meta-analyses have revealed no backfire effects among audiences who may otherwise be distrustful of experts or climate science (Rode et al., 2021; van Stekelenburg et al., 2022); and, finally, (d) the scientific consensus is an example of ethical persuasion in which the communicator is merely highlighting correct information about the level of agreement within an influential referent group instead of intending to persuade by other means.

## Conclusion

In sum, we agree with Constantino and colleagues about the power of social norms to facilitate the societal-level changes required to tackle climate change. The behavior of billions of people around the world is governed by information about what other people do and what they should be doing. We introduced another example of the ethical use of social descriptive norms, which includes highlighting the near-unanimous scientific consensus on climate change that has shown promise in changing attitudes about climate change across the political spectrum. But as Constantino et al. point out, normative power is maximized when descriptive and prescriptive norms are aligned. The challenge therefore lies in turning scientific into social consensus. This challenge is more likely to be overcome when heeding the critical considerations we have outlined here that might otherwise threaten the effectiveness of social-norm interventions, including the need to consider the adversarial, misinformation-rich environments in which normative information is communicated, the role of pervasive misperceptions about norms and the behavior of other people, the possibility that community norms can unravel quickly following key political events, and the fact that there are important differences in how susceptible people are to social influence.

## Transparency

*Editor:* Nora S. Newcombe
